# Beyond traditional surveillance: applying syndromic surveillance to developing settings – opportunities and challenges

**DOI:** 10.1186/1471-2458-9-242

**Published:** 2009-07-16

**Authors:** Larissa May, Jean-Paul Chretien, Julie A Pavlin

**Affiliations:** 1 The George Washington University, Department of Emergency Medicine, 2150 Pennsylvania Avenue, NW Suite 2B, Washington, DC 20037, USA; 2 Division of Preventive Medicine, Walter Reed Army Institute of Research, Silver Spring, M.D, 503 Robert Grant Avenue, Silver Spring, MD 20910, USA; 3 Division of Health Sciences Informatics, Johns Hopkins University School of Medicine, Baltimore, M.D, USA; 4 Global Emerging Infections System, Armed Forces Research Institute of Medical Sciences, Bangkok, Thailand, U.S. Army Medical Component, 315/6 Rajvithi Road, Bangkok 10400, Thailand

## Abstract

**Background:**

All countries need effective disease surveillance systems for early detection of outbreaks. The revised International Health Regulations [IHR], which entered into force for all 194 World Health Organization member states in 2007, have expanded traditional infectious disease notification to include surveillance for public health events of potential international importance, even if the causative agent is not yet known. However, there are no clearly established guidelines for how countries should conduct this surveillance, which types of emerging disease syndromes should be reported, nor any means for enforcement.

**Discussion:**

The commonly established concept of syndromic surveillance in developed regions encompasses the use of pre-diagnostic information in a near real time fashion for further investigation for public health action. Syndromic surveillance is widely used in North America and Europe, and is typically thought of as a highly complex, technology driven automated tool for early detection of outbreaks. Nonetheless, low technology applications of syndromic surveillance are being used worldwide to augment traditional surveillance.

**Summary:**

In this paper, we review examples of these novel applications in the detection of vector-borne diseases, foodborne illness, and sexually transmitted infections. We hope to demonstrate that syndromic surveillance in its basic version is a feasible and effective tool for surveillance in developing countries and may facilitate compliance with the new IHR guidelines.

## Background

All countries, whether high or low resourced, need sensitive disease surveillance systems for the early detection and monitoring of outbreaks. Syndromic surveillance, or the use of near "real-time" data and automated tools to detect and characterize unusual activity for further public health investigation, has been used in the United States and many other countries to augment traditional surveillance. For the purposes of this debate, we propose an expanded definition of syndromic surveillance to include the use of data on pre-diagnostic clinical syndromes rather than confirmed cases of specific diseases. The use of pre-diagnostic data and statistical algorithms aims to detect epidemics earlier than traditional surveillance based on reporting from laboratories and healthcare facilities, including atypical presentations of severe disease [1]. In 2003, over 100 different US health jurisdictions used syndromic surveillance to augment their public health surveillance [2]. In addition, several countries have used syndromic surveillance for the early detection and response to diseases of public health importance.

Despite this widespread use, syndromic surveillance is meant to enhance rather than replace traditional surveillance. An Institute of Medicine study concluded that a balance is needed between strengthening the proven approach of traditional surveillance and innovative surveillance systems [3].

In many developing countries, surveillance is limited due to the lack of a robust public health or laboratory infrastructure; however, the revised International Health Regulations [IHR], which entered into force for 194 World Health Organization [WHO] member states in 2007, require the reporting of public health emergencies of potential international concern even if the disease agent is unknown, such as for a previously unknown disease or a known disease presenting in more severe form [1,4]. Syndromic surveillance, just as in the developed world, can augment traditional surveillance in developing countries [1].

Syndromic surveillance often involves automated electronic reporting and statistical algorithms that sometimes require a complex information technology infrastructure. However, syndromic surveillance does not need to be highly computerized or technical; its tools can be simple, using few technological or human resources, and can complement existing surveillance programs [5].

An early example of "low technology" syndromic surveillance is the use of acute flaccid paralysis (AFP) as the syndromic flag for poliomyelitis. The syndrome is infrequent and may detect an excess of cases of poliomyelitis in a timely fashion by comparing observed rates of AFP to expected rates [6,7]. Nonetheless, syndromic surveillance can detect outbreaks of disease that do not fall into current WHO case classifications, which is particularly important for emerging diseases, or diseases with severe clinical presentations with undetermined diagnoses, such as the Severe Acute Respiratory Syndrome [SARS] outbreak of 2002–2003.

Since resources for surveillance are scarce in many countries, compounded by high rates of staff turnover and difficulties with Internet access and other communication tools, syndromic surveillance systems in low resourced countries need to be simple and build on prior work. The WHO open-source systems for surveillance are accessible to countries and technological assistance can be provided [5].

This paper will review the background for the IHRs and their application to syndromic surveillance, and review examples of syndromic surveillance programs that are currently being used in developing countries.

### The revised WHO International Health Regulations and Surveillance for Syndromes

Given the recent concern for pandemics such as SARS and highly pathogenic avian influenza [H5N1], global and regional surveillance should be built on the concept of integrated surveillance. Prior to their revision, the IHR mandated reporting of only three diseases to the WHO: cholera, plague, and yellow fever. A revision of the IHR undertaken in 1995 was finalized in 2005 [4]. The revised IHR address the need for strengthening of disease surveillance by modifying disease lists to include syndromes for diseases of epidemic potential, and recommend the establishment of mechanisms for reporting outbreaks of major public health importance and the development of early warning surveillance systems. The IHR now include reporting by all countries for poliomyelitis, smallpox, human influenza caused by a new subtype, SARS, cholera, plague, yellow fever, viral hemorrhagic fevers, West Nile virus [WNV], and other diseases of regional concern such as meningococcal disease and dengue [4,8].

**Figure 1:**
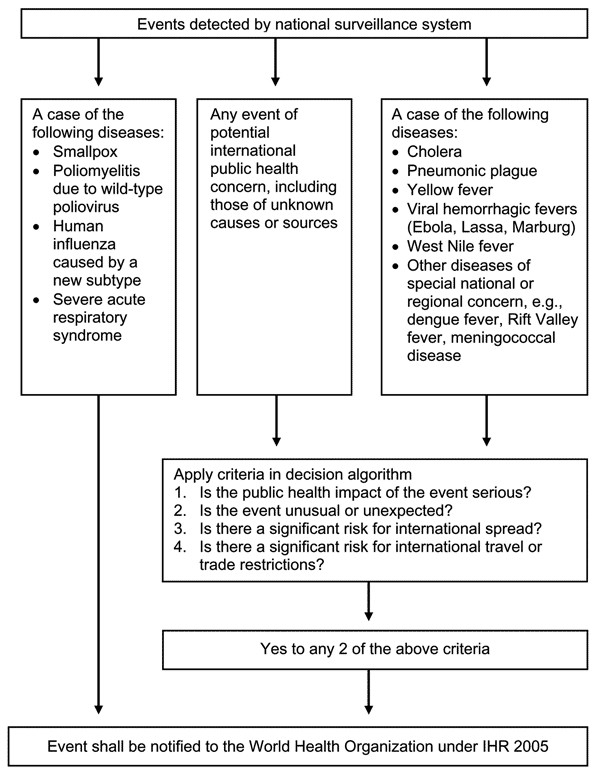
**International Health Regulations 2005 Decision Instrument**.

Annex I, part A.4 (a) of the revised IHR states that "state parties are to develop the capacity to detect events involving disease or death above expected levels for the particular time and place in all areas within the territory of the State Party," providing impetus for countries to improve their broad based public health surveillance infrastructure. IHR 2005 expands upon the previous IHR by broadening the scope of public health reporting, demanding improved surveillance and response at the country level, and strengthening core surveillance and outbreak response capacity [9]. Refer to Figure 1: International Health Regulations 2005 Decision Instrument for details.

Recommended syndromes for surveillance include hemorrhagic fever, acute respiratory syndrome, acute gastrointestinal syndrome, neurological syndrome, and a provision for severe infectious illness [10]. Although these syndromes are not officially part of the decision instrument, implementation of the syndromic approach complements the disease-specific approach with a precise definition for each syndrome, and was pilot-tested in 21 countries [8,11]. Development and field testing of syndromic reporting initially identified 5 syndromes of potential public health importance. After the interim review, WHO concluded that syndromic reporting could be useful at the country level but was not feasible for the purposes of global public health reporting due to the challenges of field reporting of syndromes and inability to standardize rules for outbreak control for these syndromes [8,11]. Baker and Fidler raised concerns that syndromic surveillance may not be effective in the timely detection of emerging diseases [9]. Although these critiques raised valid concerns, in some areas syndromic surveillance systems have detected outbreaks in a timely fashion, complementing traditional surveillance. The WHO also supported the use of syndromic surveillance at the national level during its interim review as part of strengthening core surveillance capacity. Many countries have already implemented surveillance systems to comply with the revised IHR, including surveillance for severe diarrhea, dengue fever [DF] and dengue hemorrhagic fever [DHF], and acute flaccid paralysis [e.g., refs. [7,8,12]].

A meeting of the Pan American Health Organization on the Surveillance Network for Emerging Infectious Diseases in Amazon countries in 2005 recommended the development of early warning systems, adopting the syndromic approach to surveillance to heighten the sensitivity of disease detection and improve clinical management of cases, such as the febrile icteric syndrome for yellow fever. Although in 2005 there were few guidelines for syndromic surveillance in the region, a recommendation to disseminate protocols was made [13], and new applications have since been developed, as we discuss below.

### Review of Specific Applications of Syndromic Surveillance That May be of Use in Developing Countries

#### General Surveillance for Infectious Diseases

Many syndromic surveillance systems detect general febrile illnesses such as malaria, dengue fever, other vector-borne diseases, and foodborne illness. Collaboration between countries with experience in syndromic surveillance and low resource nations has resulted in the introduction of syndromic surveillance to those countries.

The Early Warning Outbreak Recognition System [EWORS] is a collaboration between the Indonesian Ministry of Health and the US Naval Medical Research Unit-2 [NAMRU-2], in Jakarta, and was adopted by the Government of Indonesia as a national surveillance system in 2007 [1,5,14]. EWORS includes patients presenting to public hospitals and suspected of having an acute infection. Participating hospitals, clinics and emergency departments use a short, standardized questionnaire to collect demographic and clinical information. The questionnaire is filled out on a computer terminal with EWORS software and the data files are sent by email to the EWORS hub in the Ministry of Health for analysis daily. Counts of sign and symptom combinations that may reflect infectious diseases of national importance are compared to baseline counts by automated algorithms and monitored at reporting hospitals and Ministry of Health offices. If an outbreak is suspected, Ministry of Health staff may initiate investigation or control efforts. The EWORS hub also sends a monthly report to each participating site summarizing their surveillance data. System advantages include rapid data acquisition and interpretation by hospital operators, which may allow for earlier case finding. One limitation of EWORS has been the challenges with linking suspected outbreaks to response actions, which requires coordination of local bureaucracies. A second limitation is challenges with standardization of procedures at hubs, which can create alert uncertainty [15]. EWORS has now expanded to include other Southeast Asian nations, including Laos. In Peru, the Ministry of Health and US Naval Medical Research Center Detachment [NMRCD], in Lima, have collaborated to develop a similar system for early warning of dengue epidemics [15].

A second Peruvian system, Alerta, developed by Voxiva, the Peruvian Navy, and NMRCD, allows real time transmission via mobile telephone, text message, or Internet of health information from sailors and their families [1]. The system monitors all nationally notifiable diseases and syndromes, as well as other diseases of particular importance to the Peruvian Navy. [15]. Demographic and clinical data for suspected or confirmed cases of disease and syndromes is collected by the medical officer at each site, who spends ten to thirty minutes daily in medical record review for reporting. The data is then transmitted to the Alerta Disamar central hub by Internet, toll free telephone, or radio. Reporting frequency varies by disease and ranges from daily or twice weekly in batches for common syndromes such as diarrhea or respiratory illness. The hub uses Voxiva software to convert data reported by different communication methods to a common format. Staff review graphs of weekly counts automatically generated in Excel. Alerta has identified over 31 disease outbreaks [15,16] and has facilitated investigation of diarrheal disease, malaria, and influenza as well as detected an outbreak of cyclosporiasis at a naval base in Lima, Peru. Alerta has been especially useful in helping the Peruvian Navy identify and respond to outbreaks at remote bases, which previously may have gone unreported or identified long after they began because usual reporting channels were slow [15,17].

### Malaria

Syndromic surveillance may provide a relatively inexpensive tool for early detection of malaria outbreaks in low resource countries. In Ethiopia, weekly malaria cases collected from health centers in 10 districts from 1990 to 2000 were reviewed [18]. Four types of alert threshold algorithms were compared by plotting a curve for each type of alert. The curve demonstrated potentially prevented disease cases versus the number of alerts over a decade. This study found that simple weekly percentile cutoffs were as good as the more complex algorithms for detection of malaria outbreaks [18], exemplifying that syndromic surveillance can be basic while still providing useful information. WHO has advocated alerts when weekly cases exceed 75% of baseline [19]; syndromic surveillance may be able to provide early alerts of this type that will allow timely spraying and mass drug administration. The use of these types of comparative statistics in surveillance is a novel method for evaluation of the performance of malaria early warning systems [18].

In 2002, the Uganda Ministry of Health piloted a new district level monitoring system in the southwestern highlands. Incoming clinical data from health centers are collated and entered onto a district level computer and compared with a baseline of historical illness data. An anomaly measure is used to provide the index of deviation, followed by electronic reporting. This simple system detected two malaria outbreaks in Kabale, in 2005 and 2006, more than two weeks before case numbers began to peak [20].

Some surveillance systems monitor climatic and environmental data to forecast infectious disease outbreaks. In some areas, climate variables monitored by satellite can provide a two to three month lead-time for malaria epidemics [21]. In Eritrea, monthly outpatient cases of malaria in 242 districts and NDVI and rainfall datasets showed strong correlation, but coverage of clinical data stations was considered too poor to be of use in epidemic control [21]. In Tanzania, analysis of two malaria seasons in the highlands showed an association between regional rainfall and malaria cases. An early warning system based on rainfall observations may thus be useful for malaria epidemic prediction in some areas [21,22]. An example of such a system is the USAID Africa Data Dissemination website FEWS-NET, which uses rainfall-based indicators to predict change in malaria risk [23]. Similar dissemination websites could be employed in low to medium resource countries for malaria and other vector-borne diseases where remote Internet access is available.

Syndromic surveillance for malaria may enhance public health response. Since a locally transmitted case of malaria occurred in 2006 in Jamaica, active fever surveillance has been implemented for early detection at sentinel healthcare sites, airports and seaports [24]. Analysis occurs at the local level and then is transmitted centrally on a daily basis. The information is then used to conduct active door to door surveillance of fever cases if warranted.

### Dengue

Dengue surveillance is typically conducted by the passive notification of suspected or confirmed Dengue Fever (DF) or Dengue Hemorrhagic Fever (DHF) cases and deaths. These passive systems have low specificity due to infrequent laboratory confirmation but are still useful due to their simplicity and low use of resources. Unfortunately, waiting for reporting by clinicians may lead to delays in public health action and decrease efficacy of control measures. Active surveillance may include clinician sentinel networks, active fever surveillance by community health workers, and sentinel hospital systems. The first two monitor for nonspecific viral syndrome, which may also be useful for detecting outbreaks of other diseases such as influenza or malaria. A web-based reporting system may improve reporting completeness [25]. Hospital sentinel systems monitor for severe disease and death, with immediate investigation of all hemorrhagic fever. Such systems must be complemented by laboratory-based surveillance for trends and serotypes [26].

Despite concerns with its specificity, surveillance of the fever syndrome may be useful given that fever is likely caused by dengue in endemic regions [27]. In addition to serologic surveillance, there has been great interest in syndromic surveillance for detection and control of dengue [28].

As an example, in 2004 an early warning system, 2 SE FAG, was established in French Guiana with the goal of detecting outbreaks of febrile illness in French soldiers, including dengue [29]. In 2006 the system was expanded to include 25 civilian health centers that provide surveillance on sanitary conditions. Before 2006, the only data available for dengue surveillance in French Guiana was laboratory confirmed cases. They compared the frequency and timing of detected febrile cases with the traditional surveillance system for dengue, and the sensitivity was found to be high, but specificity was low [30-32]. For this system, data on all cases of fever, suspected and confirmed cases of dengue fever, and confirmed cases of malaria by syndromic surveillance syndrome definitions (fever plus headache, myalgia, arthralgia, or retro-orbital pain) are employed. Data is collected in real-time by a medical provider seeing a patient, and information is recorded on the available platform (PDA or a computer). Data is then reported to French health authorities in Cayenne. Syndromic surveillance data is converted to a weekly format and reported to the health authorities weekly in cases of normal operation, or immediately in the case of an alarm. Automated alarms are issued from the syndromic surveillance system based on current past experience graph (CPEG). Under ideal circumstances, there is a 60-minute delay between case presentation and detection by the system [30,31,33]. To evaluate the effectiveness of the system, during an outbreak of DEN-2 DF beginning in November 2006, data on confirmed dengue fever cases from a reference laboratory and data from 2SE FAG for the occurrence of undiagnosed fever associated with headaches, arthralgias, myalgias, or retro-orbital pain were compared. Levels of alarm and public health actions taken were also recorded. The syndromic surveillance pre-alarm activated 6 weeks prior to the full alarm in week 2 of 2006 and provided early warning for military personnel in comparison to the laboratory-based program [33].

The system was able to detect 6900 suspected dengue cases compared to 800 laboratory-confirmed cases in 2006. Although the sensitivity was high, specificity was low. Other limitations included incomplete report forms and dengue fever reporting being 67% higher than with traditional reports, most likely because traditional surveillance is limited to confirmed cases [30-32]. Despite its sensitivity, there has been some concern with using a febrile syndrome for dengue surveillance, given that of 7195 febrile cases, only 8% were confirmed to be dengue [13]. As in other applications of syndromic surveillance, other methods of surveillance must also be in place. A laboratory-based system for dengue may be more useful; yet given limited laboratory capabilities in dengue endemic areas, syndromic surveillance may provide valuable information on population epidemiology prior to laboratory confirmation [13]. Vector surveillance and control provides the earliest opportunity to avert or contain dengue epidemics, but many dengue-endemic countries lack resources for launching these programs.

### Other Vector-Borne Diseases

The IHR call for reporting of other vector borne diseases such as West Nile virus and Rift Valley fever. A surveillance system developed in the Netherlands for early detection of WNV focuses on cases presenting with neurologic diseases and includes the monitoring of hospital discharge data, trends in laboratory testing, and monitoring neurological diseases in horses [34]. Such a system could be applied to medium resource developing countries by monitoring the neurologic syndrome and neurologic disease in animals.

Similar to early warning systems for malaria, comparison of different prediction models for cutaneous leishmaniasis [CL] show a strong relationship with climatic variables and thus may be amenable to the development of an early warning system [35]. Models for CL incidence in Costa Rica may outperform models with no climatic indicators [36]. For vector-borne diseases with a clear relationship with the El Nino Southern Oscillation phenomenon [ENSO], models that use climate indicators to forecast disease risks are being developed [37]. In Australia, climate modeling has shown a sensitivity of up to 90% when combined with mosquito surveillance data to predict epidemics of Ross River virus disease [38]. However, many climate-based systems are not widely used due to the lack of published models outside testing areas. Disease modeling is often limited to discrete data sets for small areas [39]. Nonetheless, modeling of these data sets may be useful for selected syndromes in low resource regions. Monitoring of both climate triggers and vector-borne disease indicators together may increase sensitivity and specificity and also provide validation of data sources and backup for potential system failure.

### Respiratory Illnesses

Several recent emerging infectious disease outbreaks, such as SARS and highly pathogenic avian influenza [H5N1], arose in Asia. Surveillance for new respiratory illnesses is therefore crucial in this region. In many regions, electronic data exist that can assist with an automated system. In Taiwan, an emergency department based syndromic surveillance system for 189 hospitals automatically transmits electronic patient data to the Taiwan Centers for Disease Control. This system was built on existing work done in the United States in collaboration with the Realtime Outbreak and Disease Surveillance [RODS] Laboratory [40]. The goal of this system, among the first nationwide real time surveillance systems in Asia, is to detect winter and summer spikes in influenza-like illness, respiratory syndrome, and gastrointestinal illness [41]. Should another epidemic like SARS arise, this system may be able to provide early warning and notification, thus improving global surveillance of emerging infectious diseases. Such automated systems could be used in other medium resource regions for the detection of emerging viral illnesses.

### Foodborne Illness

Syndromic surveillance for foodborne illness is important given the globalization of the food supply and the morbidity caused by diarrhea in the developing world. Systems can monitor for gastrointestinal illnesses through the tracking of diarrhea and vomiting symptoms. In the United States, the RUSick 2 disease forum is a web-based forum that allows residents to report information on nausea, vomiting and diarrhea syndromes, including foods consumed, with the goal of identifying common food vehicles in gastrointestinal outbreaks. The goal of this system is to decrease the time delay with routine laboratory surveillance for food borne outbreaks. Completeness of the syndromic surveillance reports collected via the web-based forum has been found to be as effective as similar reports from phone calls to the health department [42,43]. Poison Control Center data has also been used to detect foodborne outbreaks, and found to be useful in early detection where there is no confirmatory diagnostic information available [44]. Establishment of these types of networks may be useful in areas with medium resources and good communications infrastructure.

These types of surveillance can also be applied to the developing world. In the Pacific region, there are four distinct levels of foodborne disease surveillance: no formal surveillance, syndromic surveillance, laboratory or pathogen-specific methods, and integrated food chain surveillance [45]. Vanuatu and Solomon Islands primarily use syndromic surveillance. Few countries have specialized laboratory surveillance, and thus information on specific pathogens is limited. A regional approach under the Pacific Public Health Surveillance Network would include development of uniform case definitions for reporting as the basis for syndromic surveillance and facilitate centralized data collection and sharing [45].

Incidence estimates of typhoid fever in Egypt have been derived recently from hospital-based syndromic surveillance along with lab-based surveillance. Although assisted by the syndromic surveillance system, the majority of patients were evaluated in the primary care system and would not have been detected by the hospital based syndromic surveillance. This situation emphasizes the ability of syndromic surveillance to augment but not replace traditional surveillance. Syndromic surveillance must be broad in scope in order to catch mild disease and expand surveillance beyond hospital data, which tends to capture more severe cases [46]. This type of surveillance may be difficult to enact in low resource regions with limited access to laboratory facilities.

As for vector-borne diseases, environmental parameters may be useful for early detection of food or waterborne disease outbreaks. One study found strong correlation between cases of *Vibrio cholera *O1 in children in Bangladesh and temperature and rainfall two to four months prior. This type of model could be a good model for a climate-based early warning system for cholera in this region [47] and could be implemented in low resource countries.

Syndromic surveillance systems have also been useful in detection of diarrheal outbreaks. EWORS facilitated the detection of a large cholera outbreak in Indonesia [48].

### Syndromic Surveillance for Sexually Transmitted Infections [STIs]

The WHO recommends a global health sector strategy as part of a multisectoral approach to responding to epidemics of STIs, including a syndromic approach for the detection and management of abnormal vaginal discharge [49]. The WHO strategy aims to decrease the cost of testing and to improve treatment practices, with a certain minimum data required for surveillance. This plan mandates knowledge of the prevalence of specific agents and their susceptibilities, which necessitates at least periodic laboratory surveillance [50]. Vaginal discharge and urethritis are the most common syndromes [51]. Syndrome case reports may include genital ulcers, urethral discharge, and vaginal discharge [52]. Monitoring of these syndromes may allow improved public health response for countries with low resources, for which automated reporting is not easily implemented. Since the introduction of syndromic surveillance in 1996 in Burkino Faso, the prevalence of STIs such as gonorrhea, syphilis, and chlamydia has decreased [53], providing an impetus to continue this surveillance. In Cote d'Ivoire, the national health information system collects data in all community-based public health clinics, which are located in 29 districts and 10 regions [54].

In the United States, the Philadelphia Department of Public Health monitors chief complaint and discharge data from emergency departments containing reportable disease information to detect cases of syphilis and manage them according to CDC guidelines [55]. These types of systems could be implemented in the developing world where electronic data or computerized systems are available.

## Summary

Syndromic surveillance is thought by many to be a high technology tool. But surveillance of syndromes is not a new phenomenon, with one of the earliest examples being acute flaccid paralysis for detection of poliomyelitis outbreaks [6]. Surveillance of influenza like illness worldwide is another example of syndrome rather than disease specific surveillance [56]. While syndromic surveillance is augmenting traditional surveillance in the developed world, it also has the potential to improve timely detection of infectious disease outbreaks in developing countries, most of which lack access to a strong public health infrastructure and specialized laboratories. The burden of public health surveillance in under-resourced and understaffed settings is a challenge. Despite this, there are several examples of low cost syndromic surveillance programs that may enhance global public health. For example, community based programs that employ volunteers may lessen the burden on hospital workers [15]. Increased use of automated reporting may decrease the burden on health care and public health workers and allow for more complete reporting of potential cases of public health importance.

Syndromic surveillance may be especially useful for early epidemic control of certain vector borne diseases as well as for diseases of public health importance that have the potential to cross international boundaries. Examples of current applications of syndromic surveillance in developing countries are summarized in Table 1. The IHR mandated the reporting of diseases of international importance; surveillance for syndromes may facilitate compliance with this IHR requirement. There is currently no infrastructure in place to enforce these guidelines, and each country must design a national surveillance system that can allow for timely detection and notification of these disease outbreaks. Although WHO is required to assist countries in developing capacity to meet these requirements, no funding is allocated from WHO for this purpose. Thus, improving countries' national public health infrastructure and reporting capabilities will require large financial and technical support, likely from countries with existing automated reporting [9]. Improvements in the communication infrastructure, including Internet access, will need to occur to allow electronic communication and enhance the timeliness of reporting. Furthermore, investment in training in epidemiology, field investigation, and information technology are vital to the future success of broader surveillance activities.

**Table 1:** Examples of Syndromic Surveillance Systems in Developing Countries

**Type of surveillance**	**Country**	**Type of Data**	**Data collection and recording methods**	**Data centralization methods**	**Analysis Frequency**	**Aberration Detection Method**	**Potential and limitations of the system for early detection of outbreaks**
Malaria	Uganda	Incidence rates	Health facilities	District level	Weekly	Anomaly measure provides index of deviation from expected weekly incidence rates	Early detection documented [20]
Malaria	Eritrea	Outpatient cases and climate datasets	242 districts via computerized access database	Central database	Monthly	Principal component analysis/non-hierarchical clustering	2–3 month lead time of peak malaria Climate variables only accurate in El Nino years [21]
Malaria	Jamaica	Active fever surveillance	Fever cases recorded at sentinel sites	Analysis at local level, then transmitted centrally	Daily then decreased over time	Not available	Active door to door surveillance [24]
Dengue Fever "2SE FAG"	French Guiana	Fever, dengue fever and malaria cases	Collected by medical provider at individual sites Recorded on IT system with syndromic software	Reported to French health authorities	Data converted to weekly format Reported immediately in case of alarm, weekly in normal operation	Automated alarm based on current past experience graph (CPEG)	Potential: 60 minutes between case presentation and system detection Improved detection of dengue Limitations: Sensitivity high but specificity low [30,31,33]
Foodborne disease	Egypt	Hospital based syndromic surveillance	Case reports	Passive reports from hospital providers	Passive surveillance	Not available	Limitations: Missed outpatients compared to laboratory surveillance [46]
Food-borne disease	Pacific Island Countries and Territories	Varies: reports of diarrheal disease; laboratory surveillance	Data collected by health care providers, reporting of laboratories	Pacific Public Health Surveillance Network to organize resources and facilitate centralized data collection and sharing	Monthly reports	Not available	No laboratory surveillance in use except for Samoa [45] Limitation: No uniform definition for foodborne disease
STI's	Burkina Faso	Prevalence studies, sentinel surveillance, population based surveys	Various methods	Not available	Not available	Not available	Decrease in incidence of gonorrhea, chlamydia and syphilis [53]
STIs	Ivory Coast	Data from three STI syndromes	Community and public clinic and hospital data computerized at district level, compiled at regional level	Data collated by districts and region then centralized nationally	Monthly	Annual incidence rates	Data provide trends of STI's and are used to estimate quantity of drugs[54]
Various Diseases: Alerta DISAMAR	Peru, operated in conjunction DOD-GEIS	Suspected or lab-confirmed cases of diseases/syndromes	Medical record review for reporting	Medical officer transmits site data to Alerta DISAMAR central hub	Daily or twice weekly	Voxiva software converts data to common format Graphs of weekly counts	Identified over 31 disease outbreaks [15,16]
Various Diseases EWORS (Early Warning Outbreak Recognition System)	Southeast Asia and Peru	Standardized questionnaire at clinical sites	Questionnaire filled out on computer terminal with EWORS software	EWORS data files sent by email to EWORS hub for analysis	Once daily; monthly report to each participating hospital Varying degrees of centralization	Automated statistical outbreak detection algorithm	Potential: detection of large cholera outbreak in Indonesia [48]; Limitations: mechanisms for linking suspected outbreaks to response; lack of standardization of procedures (15)

Although syndromic surveillance can provide useful early warning of diseases such as malaria, there are no guidelines for what to do with the information provided. It is still necessary to have a robust public health infrastructure for investigation of cases and implementation of an effective control program as with any surveillance system. Simple monitoring tools can facilitate effective epidemic control, but require the translation of this early warning information into timely public health action.

Syndromic surveillance systems should build on existing public health surveillance infrastructure, as well as work that has been done in other regions. The collaboration between the Taiwan Centers for Disease Control and the RODS Laboratory is one such example [40,41]. With increasing access to the Internet, and decreased cost and improved user friendliness of information technology in developing countries [1], novel applications for syndromic surveillance are enhancing traditional surveillance and will hopefully continue to improve the detection of outbreaks worldwide, fulfilling the goals of the IHR. We hope this review demonstrates both the effectiveness and feasibility of "low-tech" syndromic surveillance in low resources countries, and can be the starting point for further development of guidelines for how to conduct syndromic surveillance in developing countries.

## Competing interests

The authors declare that they have no competing interests.

## Authors' contributions

LM and JPC conceived the paper. LM, JPC, and JAP contributed to the design, literature review, and drafting of the manuscript. All authors read and approved the final manuscript

## Pre-publication history

The pre-publication history for this paper can be accessed here:


